# Postoperative discomfort following inverted periosteal pedicle graft versus subepithelial connective tissue graft for treating gingival recession RT1 & RT2: a randomized clinical trial

**DOI:** 10.1186/s40902-025-00499-0

**Published:** 2026-01-22

**Authors:** Marwa Elsayed, Mona Salaheldin Darhous, Ayat Gamal-AbdelNaser, Ahmed Reda Abdel Rahman

**Affiliations:** 1 Ahram Canadian University, Giza, Egypt; 2 Cairo University, Cairo, Egypt; 3 October University of Modern Sciences and Arts, Giza, Egypt

**Keywords:** Periodontal plastic surgery, Regeneration, Surgical papillae, Vestibular deepening

## Abstract

**Background:**

Despite being the most predictable root coverage technique, coronally advanced flap with subepithelial connective tissue graft (CAF + SCTG) necessitates graft harvesting from a second surgical site; increasing patient’s post-operative discomfort. Besides, due to the promising regenerative capacity of the periosteum and omitting the need for a second surgical site, periosteal pedicle graft (PPG) is tested as an alternative technique. Eighteen RT1 and RT2 gingival recession defects were randomly assigned to receive either CAF + SCTG or inverted PPG (iPPG). Postoperative discomfort, recession depth, and patient’s satisfaction were assessed within 6-months of follow-up.

**Results:**

Patients in the iPPG group reported postoperative discomfort (VAS) of (2.28 ± 0.83) compared to (3.11 ± 1.17) in SCTG group; with no statistically significant difference. Clinically, the mean recession depth was significantly decreased from baseline in iPPG group (from 3.78 ± 0.67 to 0.56 ± 0.53) and in SCTG group (from 3.89 ± 0.78 to 0.33 ± 0.5). Lastly, iPPG showed significantly higher level of patients’ satisfaction compared to SCTG.

**Conclusions:**

iPPG can be a promising alternative to SCTG for root coverage with significantly increased patient’s satisfaction after six months and comparable clinical root coverage parameters.

## Introduction

Gingival recession represents a widespread clinical symptom that follows periodontal tissue damage [1]. If untreated, gingival recession does not improve spontaneously and rather proceeds with time [2]. Although coronally advanced flap with subepithelial connective tissue graft (CAF + SCTG) is considered the gold standard for gingival recession surgical treatment, the procedure has its inherent drawbacks [3]. Graft harvesting from a second surgical site contributes to increased postoperative morbidity and prolonged surgical procedures. Besides, limited graft tissue availability might not suffice large defects [2].

To overcome these shortcomings, the periosteum was proposed as a substitute to connective tissue grafts. Other than being autogenous, periosteum has its own blood supply, possesses regenerative cells and fibrous content, and can be harvested from the same surgical site in sufficient amount [4].

After the first introduction of periosteal pedicle graft (PPG) [5], Steiner and associates [6] suggested that the use of PPG might not benefit from the periosteal regenerative cells when placed facing the flap tissue rather than the exposed root surface. Therefore, inverting the PPG was proposed; assuming that thereby true regeneration would be achieved. In addition, the outer fibrous layer of the periosteum overlying the regenerative cells would act as a flap support [7].

As promising as the scientific basis of the inverted PPG (iPPG) technique is in obtaining true regeneration, -to the best of our knowledge,- it was tested only in few studies, mainly case reports [4, 7, 8]. Thus, this clinical trial aims to be the first to investigate the effectiveness of iPPG compared to the gold standard (CAF + SCTG) in managing RT1 and RT2 gingival recession defects in terms of patient-reported outcome measures (PROMs) and clinical parameters.

## Materials and methods

### Settings

The current randomized, controlled, parallel-grouped clinical trial was held in the outpatient clinic of Oral Medicine and Periodontology department, Faculty of Dentistry; from November 2018 till December 2020. The protocol of the study was registered on *clinicaltrials.gov* (NCT03701191).

### Participants

The current RCT included 18 patients with gingival recession defects of RT1 or RT2 types; to whom surgical root coverage was performed.

The study included adult patients having one or more teeth with RT1 or RT2 gingival recession defects; that were not previously treated by other surgical root coverage procedures. The teeth with the recession defects were included only when the gingival keratinized tissue was of a minimal width of 2 mm.

However, patients were excluded when having systemic disorders or taking medications that contraindicate surgical intervention. Besides, cases were also excluded when showing signs of: (i) acute infection or inflammation in the surgical site or the whole oral cavity, (ii) persistent uncorrected tooth brushing-induced gingival trauma, or (iii) untreated periodontitis. Furthermore, heavy smokers (> 10 cigarettes/day), pregnant females and physically and mentally handicapped patients were excluded. Lastly, the patient was excluded if the gingiva had thin biotype or the involved tooth was severely mal-posed, rotated, clinically over-erupted or has non-carious cervical lesions (NCCLs).

### Sample size calculation

The sample size for the superiority trial was calculated based on the pre-reported effect size of postoperative discomfort of 1.8 on visual analogue scale (VAS) [9]. Using 80% power and 0.05 α, independent t-test on PS software (Vanderbilt, Tennessee, USA) calculated 14 gingival recession defects (seven per group) to be included. Adding 25% to the sample to make up for anticipated attrition, the sample was increased to 18 defects (nine per group).

### Interventions

Each eligible patient received a detailed explanation of the study; and upon providing their informed consent, they were randomly allocated to either: (a) the test group to which iPPG was performed; or (b) the control group where CAF + SCTG was performed.

### Randomization and allocation concealment

Before the study, the random sequence was generated by a third party using sealedenvelope.com in the form of block randomization of block size 6 and allocation ratio 1:1. Being concealed of the operating surgeon, the type of intervention was assigned to the included patient following a phone call.

### Blinding

As the two interventions were technically different, blinding of the operator and the patient was not feasible. Thus, blinding was restricted to the two outcome assessors: (a) a periodontist who was not involved in the research and (b) a dentist of another specialty. Before recruiting the first patient, the measurement techniques of the operator and the outcome assessors were calibrated and validated.

### Study steps

After initial assessment of the case, steps 1 and 2 of periodontal therapy were performed in the form of non-surgical periodontal therapy [10]; together with patient’s education of correct oral hygiene measures. Preoperative periodontal measurements were recorded: bleeding on probing (BOP), baseline gingival recession depth (mm) and probing depth (mm). The CEJ was considered the reference point for outcomes assessment during follow-up sessions.

### Surgical procedures

**Fig. 1:**
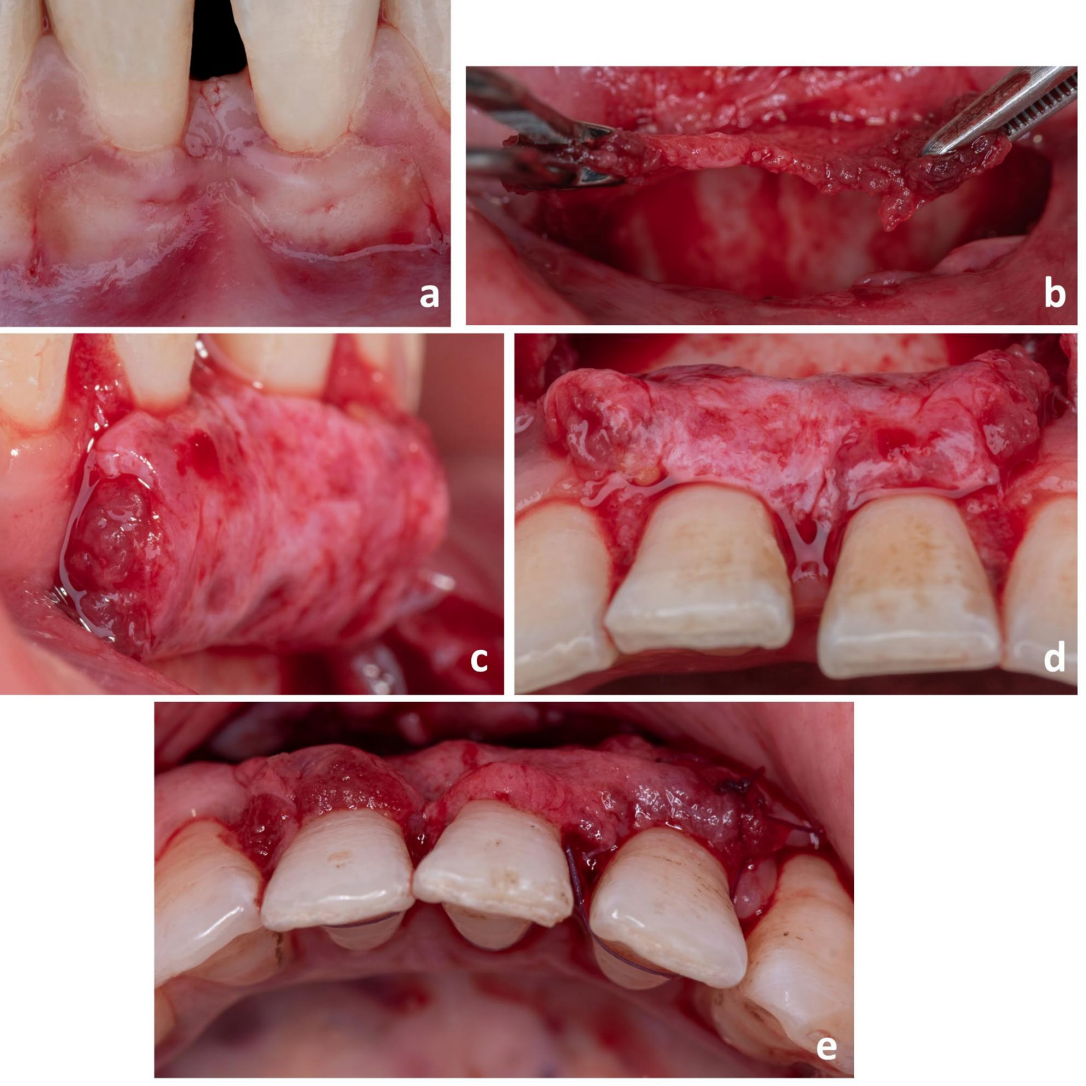
Case 1. Surgical procedures of intervention case (iPPG) including (**a**) Flap design; (**b**) Periosteal graft attached to bone at its coronal aspect; (**c**) Periosteal graft inversion (lateral view); (**d**) Inverted periosteal pedicle graft (occlusal view); (**e**) Continuous sling suture for graft fixation (occlusal view)

After local anesthesia administration, iPPG technique was performed according to the procedures highlighted by Shetty [11] with some modifications as follows (Fig. 1):

- Incisions: Horizontal incisions were made at the level of the cemento-enamel junction (CEJ) on the facial aspect of the affected tooth/teeth; followed by extending intra-sulcular incisions mesio-distally to dissect the facial aspect of the adjacent papillae while precluding the gingival margin of the adjacent teeth. Two vertical releasing incisions were made from the mesial and distal extremities of the horizontal incisions (creating surgical papillae) extending beyond the muco-gingival junction.
- Flap design: A partial thickness flap was, then, elevated till an adequate amount of periosteum was exposed (with nearly double the amount of recession exposed). Mesio-distal and apical sharp dissection parallel to the vestibular lining mucosa was performed (with the blade applied parallel to the mucosa), till passive coronal flap displacement was accomplished. The flap was checked to be stable in its final coronal position, even without the sutures.

At the apical extent of the periosteum sufficient to cover the denuded root surface, a horizontal incision was performed, while it was still attached to the underlying bone. Two other vertical incisions were made in the periosteum coincident with the flap vertical incisions, but not reaching the coronal horizontal incisions (Fig. 1a).

Periosteum dissection and inversion: The periosteum was then bluntly dissected from the underlying bone using a thin mucoperiosteal elevator. Dissection started from the apical horizontal incision performed in the periosteum and progressed coronally. The periosteum was totally dissected from the underlying bone, except for its coronal segment that was left attached to bone (the periosteal graft) (Fig. 1b).

The papillae adjacent to the involved tooth/teeth (anatomical papillae) were de-epithelialized. The reflected periosteum was, then, inverted such that the cambium layer (inner layer) covered the denuded root surface (Figure 1c and d).

- Suturing: Once the periosteum was in the required position at the level of CEJ, it was secured using 5 − 0 absorbable vicryl suture material, where a continuous-sling suture was applied. Interrupted sutures were also done in some cases on mesial and distal aspects. Interrupted 5 − 0 polypropylene sutures were performed along the vertical incisions of the split-thickness flap, starting from its apical corners and proceeding coronally, to ensure passive coronal flap advancement. Sling 5 − 0 polypropylene sutures anchored around the lingual/palatal cingula of involved teeth were then applied, to stabilize the flap in its coronal position while covering the periosteal graft completely and securing the surgical papillae over the de-epithelialized anatomical papillae (Fig. 1e).

For the control group, the CAF procedure was carried out following De Sanctis and Zucchelli [12] trapezoidal design and SCTG was harvested using trap-door technique as described by Edel [13].

### Postsurgical instructions and infection control

To control pain, 600 mg ibuprofen tablet was prescribed before the procedures and repeated 6 h post-operatively. Patients were also directed to eat soft diet, avoid local trauma and discontinue tooth brushing in the surgical site only during the first three weeks postoperatively. Local disinfection was achieved by prescribing 0.12% Chlorhexidine-based rinses for 1 min three times daily. Sutures were removed 14 days postoperatively.

### Outcomes

**Fig. 2:**
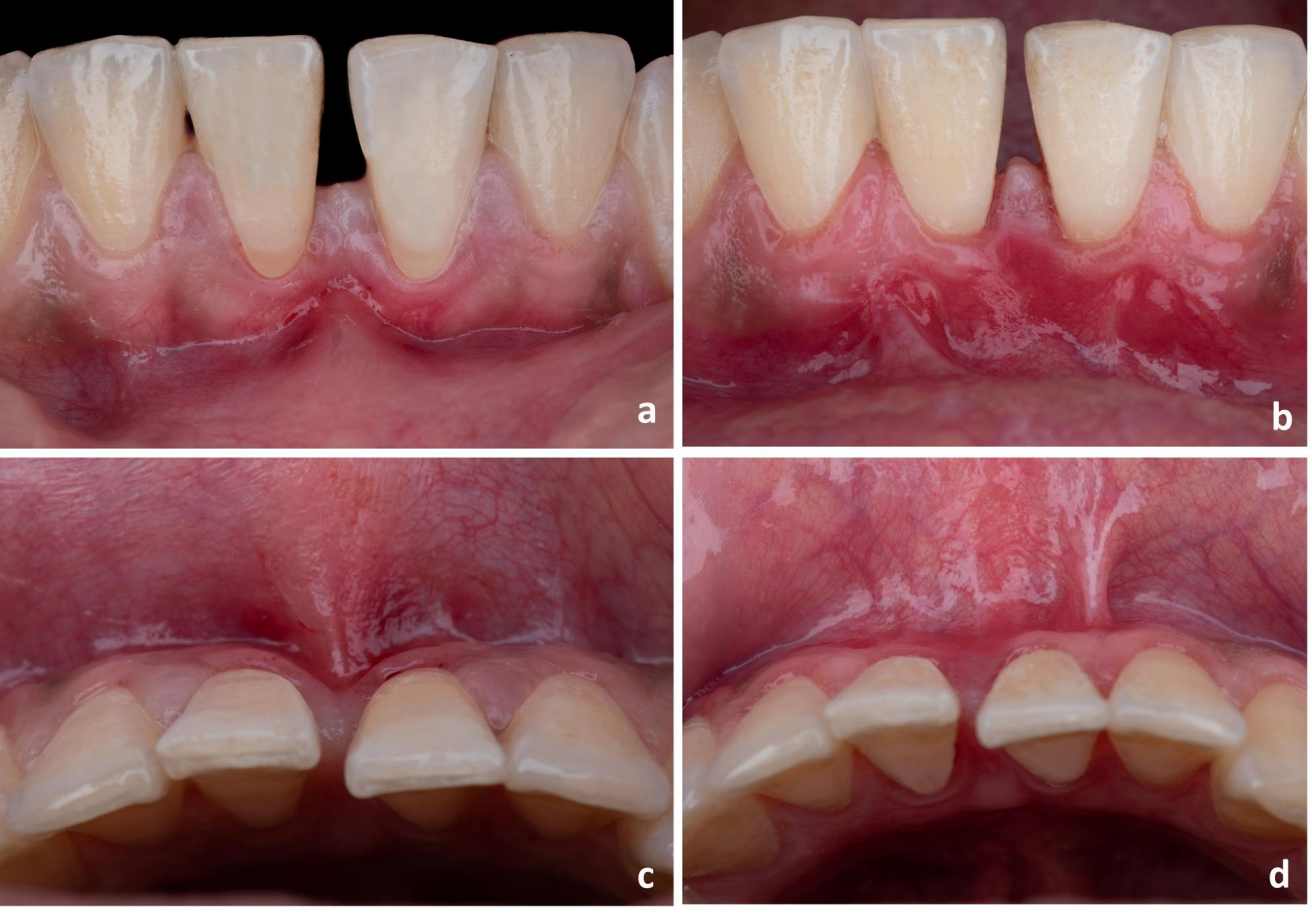
Case 1 (Before-and-after): (**a**) Baseline (frontal view) (**b**) After 6 months (frontal view) (**c**) Baseline (occlusal view) (**d**) After 6 months (occlusal view)

**Fig. 3:**
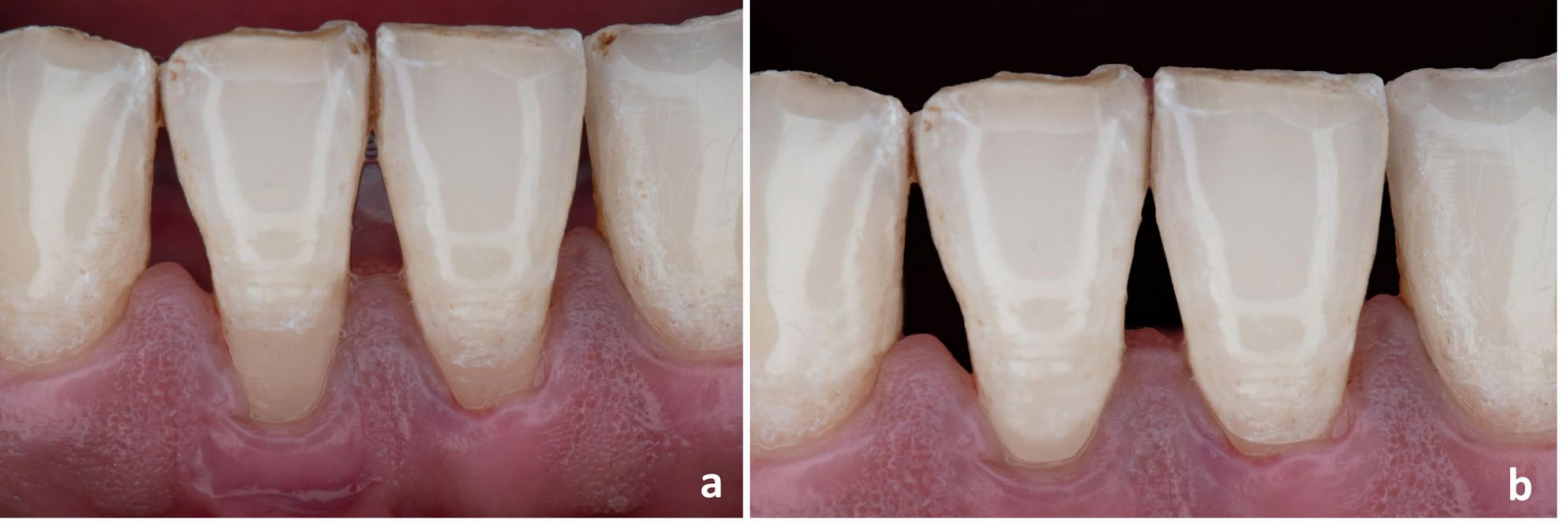
Case 2 (Before-and-after): (**a**) Baseline (**b**) After 6 months

Patients were followed up for a period of 6 months after root coverage procedure, during which the outcomes were assessed (Figs. 2 and 3). The primary outcome was postoperative discomfort; assessed directly on intervention day and after two weeks using VAS [9].

The secondary outcomes included: recession depth calculated in mm [14] assessed at three months and six months postoperative time and; patient’s satisfaction (using 3-points rating scale [15]) evaluated at the end of the follow up period.

Recession depth was assessed by William’s graduated probe as the distance from CEJ to the gingival margin at the deepest point of recession of the affected tooth; while root coverage was calculated by subtracting the recession depth at three months and at six months from the recession depth at baseline [16].

Patient satisfaction questionnaire included seven criteria that the patient was asked to evaluate on a 3-point scale with 1 indicating “unsatisfied”, 2 meaning “satisfied” and 3 denoting “fully satisfied”. Patients expressed their satisfaction level regarding the following criteria: attained root coverage, relieved dentinal hypersensitivity, gingival color, gingival shape and contour, surgical procedures (pain during surgery and the discomfort experienced related to the duration of the procedure and handling by the operator), post-surgical phase (pain, swelling, and postoperative complications) and cost-effectiveness (whether the improvement felt justifies the time and money spent for treatment) [15]. The scores of all criteria were then added to calculate the total satisfaction score for each individual. Total scores ranging between 1 and 7 are considered “unsatisfied”, those from 8 to 14 are regarded “satisfied” and a range of 15 to 21 is considered “fully satisfied”.

### Statistical analysis

Statistical analysis was performed by SPSS (version 20). Fisher’s exact test was used for categorical variables contingency tables. For comparing two groups mean of numerical quantitative variables, the independent samples t-test was applied. For more than two groups analysis of variance, ANOVA test was used to compare the means followed by post hoc multiple comparison test using Bonferroni method. After ensuring normality by Shapiro-Wilk test, paired t-test was used for intragroup analysis of two points of time; and for more than two points, Friedman test was applied. Significance level was considered at *P* < 0.05. Two-tailed tests were assumed throughout the analysis for all statistical tests.

## Results

The current study included 18 participants having RT1 or RT2 gingival recession defects allocated equally into test (iPPG) group or control (SCTG) group. The treated defects were all located at the mandibular anterior region. Healing of all participants was uneventful. They all showed compliance to the study till the last follow up.

### Baseline data

The 2 groups included participants who were not significantly different regarding their age and sex; while baseline clinical data of recession type (RT) showed higher percentage of RT2 defects in the test group compared to the control group (Table 1).

**Table 1:** Baseline demographic and clinical data of the included participants

	Age (Mean (SD))	Sex (*n* (%))		Recession type (RT) (*n* (%))	
		Males	Female	RT1	RT2
iPPG Group (*n* = 9)	31.33 (6.42)	2 (22.22)	7 (77.78)	1 (11.11)	8 (88.89)
SCTG Group (*n* = 9)	36.22 (8.64)	3 (33.33)	6 (66.67)	6 (66.67)	3 (33.33)

### Outcomes

#### Postoperative discomfort

Intergroup analysis shows absence of statistical significance of VAS scores at intervention day, 2 weeks and 3 months postoperatively (*p* = 0.49, 0.21 and 1 respectively). After two weeks, the test group participants experienced no postoperative discomfort (mean = 0.00 ± 0.00); while for control group, 0.44 ± 1.01 mean postoperative discomfort was reported. The postoperative discomfort reported by patients of the control group was related to pain at the donor site. However, Intragroup analyses showed significant reduction in discomfort scores along the follow up periods in each of the study groups (Table 2).

**Table 2:** Patient’s discomfort through the follow up period as VAS scale

	Intervention day Mean (SD)	2 weeks Mean (SD)	3 months Mean (SD)	*P*-value**
iPPG group (*n* = 9)	2.78 (0.83)	0 (0)	0 (0)	0.00*
SCTG group (*n* = 9)	3.11 (1.17)	0.44 (1.01)	0 (0)	0.00*
*P*-value†	0.49	0.21	1	

*Significant at *P* ≤ 0.05

**†**Intergroup analysis by Independent t-test

** Intragroup analysis by Friedman test

#### Recession depth

Recession depth was reduced significantly by each of the study procedures through the first six months postoperatively (Table 3).

**Table 3:** Recession depth (mm) through time in the two groups

	Recession depth (mm)				Root coverage (mm)		
	Baseline Mean (SD)	3 months Mean (SD)	6 months Mean (SD)	*P*-value**	3 months Mean (SD)	6 months Mean (SD)	*P*-value‡
iPPG group (*n* = 9)	3.78 (0.67)	1.33 (0.87)	0.56 (0.53)	0.00*	2.44 (0.53)	3.22 (0.67)	0.008*
SCTG group (*n* = 9)	3.89 (0.78)	0.78 (0.83)	0.33 (0.50)	0.00*	3.11 (0.33)	3.56 (0.53)	0.035*
*P*-value†	0.75	0.006*	0.26		0.005*	0.26	

*Significant at *P* ≤ 0.05

**†**Intergroup analysis by Independent t-test

**Tested by variance ANOVA test

**‡**Tested by paired-t-test

Comparing between the two groups at 3 months, the test group attained mean root coverage (MRC) 2.44 ± 0.53 mm, compared to 3.11 ± 0.33 mm in the control group with a statistically significant difference (*P* = 0.005). However, the difference was not statistically significant at 6 months (*P* = 0.26) (Table 3).

#### Patients’ satisfaction

By the end of the study, the patients in the test group reported statistically significant higher levels of satisfaction (Table 4).

**Table 4:** Frequencies (n) and percentage (%) of patients’ satisfaction in each treatment group

	iPPG group (*n*=9)		SCTG group (*n*=9)		*P*-value**
	Frequency (n)	Percent (%)	Frequency (n)	Percent (%)	
Unsatisfied	0	0.00%	4	44.44%	0.014*
Satisfied	2	22.22%	4	44.44%	
Fully satisfied	7	77.78%	1	11.11%	

*Significant at* P≤0.05*

**Tested by Fisher Exact probability test

## Discussion

The current study evaluates iPPG as an alternative to SCTG for treating gingival recession. This is based on technique of iPPG which eradicates the need for a second surgical site; hence reducing postoperative discomfort and duration of the surgical procedure. Besides, possessing its own blood supply is expected to provide the iPPG a more favorable healing course [15]. Furthermore, the periosteum has positive attributes including its osteogenic, regenerative capacity and possession of stem cells [17, 18].

Therefore, in the surgical procedure of the test group of the present study, the periosteal pedicle flap was inverted, to benefit from the placement of inner cambium layer with its high cellular content in contact with the exposed root surface. Thus, when placing the cells capable of formation of new bone, cementum and periodontal ligaments facing the root, it is postulated to enhance true periodontal tissue regeneration. Moreover, the outer fibrous layer is expected to function as a flap support [6, 19].

Postoperative discomfort was assigned as the primary outcome based on the consensus reports [20, 21]; especially while considering, the direct effect of PROMs on the patients’ preference for a certain root coverage procedure rather than the other [21].

On the intervention day, iPPG group reported insignificant difference in the levels of postoperative discomfort compared to the SCTG group. After two weeks of intervention, all the iPPG group participants experienced no post-operative discomfort; unlike the SCTG group in which pain related to the donor site persisted to the second week postoperatively.

Despite clinically significant, the difference between the means of discomfort scores in the two groups at baseline and after two weeks did not show statistical significance. Hence, the conclusion that “iPPG is associated with lower postoperative discomfort than SCTG on intervention day and at two weeks postoperatively” can neither be refuted nor confirmed by the results of the current study with the need for a larger sample size to be detected. However, the preliminary results favor iPPG over SCTG due to lack of donor site pain.

Shifting to the clinical parameters, recession depth showed highly statistically significant reduction in both groups during the six months. These results were in accordance with preceding clinical trials [15, 20, 22–24] which evaluated the effect of PPG as a root coverage procedure. Therefore, in the present study, the two used surgical procedures were hugely successful in managing gingival recession.

Gathering the findings, one can clearly notice the superiority of SCTG in increasing root coverage after three months. At the same time, the results of iPPG are remarkably improved at six months; to be comparable to the 6-months results of SCTG. This observation raises the hypothesis that iPPG might require longer time to achieve closer root coverage results attained by SCTG. Moreover, the delayed coverage could be attributed to the expected periodontal tissue regeneration attained by iPPG compared to healing by long junctional epithelium associated with other techniques including SCTG [7].

At the end of the follow up period of the study (after six months), patients’ overall satisfaction was assessed. None of the participants in iPPG group was un-satisfied (0.00%) versus 44% in SCTG group with statistically significant difference. Dissatisfaction was attributed to the stressful long surgical time and the pain at the palatal wound which exceeded that of the recession defect itself.

Other than the pre-specified outcomes, the operators noticed some positive clinical observations. As all (100%) defects treated by the iPPG technique were located in the mandibular anterior region and were associated with shallow labial vestibule, an obvious vestibular deepening was detected in all cases three months postoperatively. That is, the surgical procedure of iPPG was detected to inherently enable the deepening of the vestibule and increase keratinized tissue width.

Thus, beside its promising results in improving root coverage with higher patient’s satisfaction compared to SCTG, iPPG can be proposed as an alternative to indirect/two-step CAF approach for root coverage [25]; thus evading patients’ morbidity following two surgical procedures with free gingival graft (FGG) harvesting.

Although iPPG is characterized by its simple technique; yet, caution is required if performed at the mandibular premolar region, to avoid the risk of endangering the mental foramen. Also, cases of thin gingival biotype are not recommended, to enable the achievement of optimum flap and periosteal graft thickness.

The current study clarified the encouraging effects of iPPG attained through the periosteum. But being a pilot study, the sample size did not allow for a huge effect size to be detected. Furthermore, the sample size did not allow for including defects within great diversity of dental quadrants where the included defects were mainly in the most commonly affected area, namely the mandibular anterior area [26].

In the current study, RT2 recession type defects were included in addition to RT1 to evaluate the capability of the periosteum in improving the interdental recession defects in RT2. Besides, from the biologic stance, restoring the tooth surrounding periodontium (i.e. formation of new periodontal ligaments, root cementum and alveolar bone) should be as important in periodontal plastic surgeries as the restoration of the lost soft tissue architecture [27]. Nonetheless, recession defects happened to be unequally distributed in the two study groups due to mere chance by the random sequence. However, this unequal distribution is not possibly caused by or causing any bias; as the more severe form (RT2) was allocated mainly in the intervention group. Yet, the iPPG group attained comparable results to the control group which included milder defects. Also, the present study was concerned primarily with patient-centered outcomes. Therefore, further clinical trials of larger sample sizes are demanded; while assessing additional outcomes as vestibular depth, keratinized tissue width and gingival thickness.

## Conclusions

Within the limitations of the study, it could be concluded that iPPG might be a promising alternative to SCTG for root coverage with superior patient satisfaction. iPPG provided comparable results to SCTG clinically and concerning post-operative discomfort.

## Data Availability

The datasets analyzed during the current study are available from the corresponding author on reasonable request.

## Abbreviations

CAF: Coronally advanced flap
CEJ: Cemento-enamel junction
iPPG: Inverted periosteal pedicle graft
NCCLs: Non-carious cervical lesions
PPG: Periosteal pedicle graft
PROMs: Patient-reported outcome measures
SCTG: Subepithelial connective tissue graft

## References

[1] Cao Q, Lu R, Chen J et al (2021) Treatment of gingival recession with microinvasive surgical technology. J Nanomater 2021:1:9972879. 10.1155/2021/9972879

[2] Zucchelli G, Mounssif I (2015) Periodontal plastic surgery. Periodontol 2000 68:333–368. 10.1111/prd.12059 25867992 10.1111/prd.12059

[3] Zuhr O, Bäumer D, Hürzeler M (2014) The addition of soft tissue replacement grafts in plastic periodontal and implant surgery: critical elements in design and execution. J Clin Periodontol 41:S123–S142. 10.1111/jcpe.12185 24640997 10.1111/jcpe.12185

[4] Gupta GK, Kulkarni MR, Thomas BS (2014) Post-operative morbidity following the use of the inverted periosteal graft: a case series. J Indian Soc Periodontol 18:82–84. 10.4103/0972-124X.128197 24744551 10.4103/0972-124X.128197 PMC3988651

[5] Gaggl A, Jamnig D, Triaca A, Chiari FM (2005) A new technique of periosteoplasty for covering recessions preliminary report and first clinical results. Periodont Pr Today 2:55–62

[6] Steiner GG, Kallet MP, Steiner DM, Roulet N (2007) The inverted periosteal graft. Compend Contin Educ Dent 28:154–161 17385397

[7] Singh AK (2018) The periosteum inversion technique for coverage of exposed root surface of gingival recession. J Interdiscip Dent 8:127–131

[8] Ambili R, Gopakumar D, Burhan B, Badarudhin K (2024) Free gingival graft embossed over laterally flipped periosteum for root coverage: a novel case report. J Indian Soc Periodontol 28:143–146. 10.4103/jisp.jisp_447_23 38988969 10.4103/jisp.jisp_447_23 PMC11232801

[9] Cairo F, Cortellini P, Pilloni A et al (2016) Clinical efficacy of coronally advanced flap with or without connective tissue graft for the treatment of multiple adjacent gingival recessions in the aesthetic area: a randomized controlled clinical trial. J Clin Periodontol 43:849–856. 10.1111/jcpe.12590 27329829 10.1111/jcpe.12590

[10] Sanz M, Herrera D, Kebschull M et al (2020) Treatment of stage I – III periodontitis — the EFP S3 level clinical practice guideline. J Clin Periodontol 47:4–60. 10.1111/jcpe.13290 32383274 10.1111/jcpe.13290 PMC7891343

[11] Shetty N (2014) Inverted periosteal technique – a solution to multiple teeth recession. Dent Open J 1:10–13

[12] De Sanctis M, Zucchelli G (2007) Coronally advanced flap: a modified surgical approach for isolated recession-type defects: three-year results. J Clin Periodontol 34:262–268. 10.1111/j.1600-051X.2006.01039.x 17309597 10.1111/j.1600-051X.2006.01039.x

[13] Edel A (1974) Clinical evaluation of free connective tissue grafts used to increase the width of keratinised gingiva. J Clin Periodontol 1:185–196 4533490 10.1111/j.1600-051x.1974.tb01257.x

[14] Zucchelli G, Mele M, Stefanini M et al (2010) Patient morbidity and root coverage outcome after subepithelial connective tissue and de-epithelialized grafts: a comparative randomized-controlled clinical trial. J Clin Periodontol 37:728–738. 10.1111/j.1600-051X.2010.01550.x 20590963 10.1111/j.1600-051X.2010.01550.x

[15] Mahajan A, Bharadwaj A, Mahajan P (2012) Comparison of periosteal pedicle graft and subepithelial connective tissue graft for the treatment of gingival recession defects. Aust Dent J 57:51–57. 10.1111/j.1834-7819.2011.01648.x 22369558 10.1111/j.1834-7819.2011.01648.x

[16] O’LEARY TJ (1972) The plaque control record. J Periodontol 43:38. 10.1902/jop.1972.43.1.38 4500182

[17] Iyer S, Sidharthan S, Gopalakrishnan D et al (2024) Clinical efficacy of periosteal pedicle graft as a barrier membrane in guided tissue regeneration. A systematic review and meta – analysis . Dent Res J 21(1):37 PMC11346614 39188392

[18] Mahajan A, Goyal L, Asi KS et al (2023) Clinical effectiveness of periosteal pedicle graft for the management of gingival recession defects—a systematic review and meta-analysis. Evid Based Dent 24:93–94 37286696 10.1038/s41432-023-00898-0

[19] Singh N, Uppoor A, Nayak D (2015) Bone’s smart envelope - the periosteum: unleashing its regenerative potential for periodontal reconstruction. Int J Contemp Dent Med Rev 2015:1–5. 10.15713/ins.ijcdmr.62

[20] Tatakis D, Chambrone L, Allen E et al (2015) Periodontal soft tissue root coverage procedures: a consensus report from the AAP regeneration workshop. J Periodontol 86:S52–5 25315018 10.1902/jop.2015.140376

[21] Tonetti MS, Jepsen S (2014) Clinical efficacy of periodontal plastic surgery procedures: consensus report of group 2 of the 10th European workshop on periodontology. J Clin Periodontol 41:S36–S43. 10.1111/jcpe.12219 24640999 10.1111/jcpe.12219

[22] Mahajan A, Asi KS (2018) Comparison of effectiveness of the novel periosteal pedicle graft technique with coronally advanced flap for the treatment of long-span unesthetic multiple gingival recession defects. Clin Adv Periodontics 8:77–83. 10.1002/cap.10015

[23] Bhavana P, Gottumukkala SNVS, Penmetsa GS et al (2023) Clinical evaluation of periosteal pedicle flap in the treatment of gingival recessions for esthetic root coverage: A randomized controlled clinical trial. J Indian Soc Periodontol\ 27:76–81. 10.4103/jisp.jisp 36873965 10.4103/jisp.jisp_80_22 PMC9979811

[24] Maity S, Priyadharshini V, Nisha S, Shashikumar P (2022) Periosteal pedicle graft: a leading technique for the treatment of single and multiple recession defects : a case series. J Datta Meghe Inst Med Sci Univ 17:949–953. 10.4103/jdmimsu.jdmimsu

[25] Wessel JR, Tatakis DN (2008) Patient outcomes following subepithelial connective tissue graft and free gingival graft procedures. J Periodontol 79:425–430. 10.1902/jop.2008.070325 18315424 10.1902/jop.2008.070325

[26] Agusto M, Salman A, Parker D et al (2021) Root coverage predictability in the treatment of gingival recessions on mandibular anterior teeth. JDR Clin Transl Res 2380084421. 10.1177/23800844211009437 10.1177/23800844211009437 33899565

[27] Sculean A, Cosgarea R, Stähli A et al (2016) Treatment of multiple adjacent maxillary miller class I, II, and III gingival recessions with the modified coronally advanced tunnel, enamel matrix derivative, and subepithelial connective tissue graft: A report of 12 cases. Quintessence Int (Berl) 47:653–659. 10.3290/j.qi.a36562 10.3290/j.qi.a36562 27446995

